# Establishment of a core outcome set for burn care research: development and international consensus

**DOI:** 10.1136/bmjmed-2022-000183

**Published:** 2022-07-07

**Authors:** Amber Young, Anna Davies, Carmen Tsang, Jamie Kirkham, Tom Potokar, Nicole Gibran, Zephanie Tyack, Jill Meirte, Teruichi Harada, Baljit Dheansa, Jo Dumville, Chris Metcalfe, Rajeev Ahuja, Fiona Wood, Sarah Gaskell, Sara Brookes, Sarah Smailes, Marc Jeschke, Murat Ali Cinar, Nukhba Zia, Amr Moghazy, Jonathan Mathers, Sian Falder, Dale Edgar, Jane Mary Blazeby

**Affiliations:** 1 Centre for Surgical Research, Population Health Sciences, Bristol Biomedical Research Centre, Bristol Medical School, University of Bristol, Bristol, UK; 2 Division of Population Health, Health Services Research & Primary Care, University of Manchester, Manchester, UK; 3 Centre for Global Burn Injury Policy and Research, Swansea University, Swansea, UK; 4 UW Medicine Regional Burn Center, Harborview Medical Center, UW Department of Surgery, University of Washington (UW), Seattle, WA, USA; 5 Child Health Research Centre, Faculty of Medicine, Centre for Children’s Burns and Trauma Research, Queensland University of Technology, Brisbane, QLD, Australia; 6 Department of Medicine and Health Sciences, University of Antwerp, Antwerpen, Belgium; 7 Seitokai Medical and Social Welfare Corporation, Teramoto Memorial Hospital, Kawachinagano, Osaka, Japan; 8 Department of plastic surgery and burns, Queen Victoria Hospital, East Grinstead, UK; 9 Division of Nursing, Midwifery, and Social Work, University of Manchester, Manchester, UK; 10 Bristol Medical School, University of Bristol, Bristol, UK; 11 Department of Burns & Plastic Surgery, Lok Nayak Hospital and Maulana Azad Medical College, New Delhi, India; 12 Burn service, University of Western Australia, Perth, WA, Australia; 13 Paediatric Psychosocial Service, Royal Manchester Children's Hospital, Manchester, UK; 14 Institute of Cancer and Genomic Sciences, University of Birmingham, Birmingham, UK; 15 Department of physiontherapy, Broomfield Hospital, Mid Essex Hospitals, Chelmsford, UK; 16 Department of Surgery and Plastic Surgery, Sunnybrook Research Institute, University of Toronto, Toronto, ON, Canada; 17 Department of Physical Therapy and Rehabilitation, Hasan Kalyoncu University, Gaziantep, Turkey; 18 Johns Hopkins International Injury Research Unit, Johns Hopkins University Bloomberg School of Public Health, Baltimore, MD, USA; 19 Department of plastic surgery, Suez Canal University, Ismailia, Egypt; 20 Institute of Applied Health Research, University of Birmingham, Birmingham, UK; 21 Department of plastic surgery, Alder Hey Children's NHS Foundation Trust, Liverpool, UK; 22 Adult Burns Unit, Fiona Stanley Hospital, Murdoch, WA, Australia

**Keywords:** Qualitative research, Quality of health care, Surgery, plastic, Wounds and injuries, Specialties, surgical

## Abstract

**Objective:**

To develop a core outcome set for international burn research.

**Design:**

Development and international consensus, from April 2017 to November 2019.

**Methods:**

Candidate outcomes were identified from systematic reviews and stakeholder interviews. Through a Delphi survey, international clinicians, researchers, and UK patients prioritised outcomes. Anonymised feedback aimed to achieve consensus. Pre-defined criteria for retaining outcomes were agreed. A consensus meeting with voting was held to finalise the core outcome set.

**Results:**

Data source examination identified 1021 unique outcomes grouped into 88 candidate outcomes. Stakeholders in round 1 of the survey, included 668 health professionals from 77 countries (18% from low or low middle income countries) and 126 UK patients or carers. After round 1, one outcome was discarded, and 13 new outcomes added. After round 2, 69 items were discarded, leaving 31 outcomes for the consensus meeting. Outcome merging and voting, in two rounds, with prespecified thresholds agreed seven core outcomes: death, specified complications, ability to do daily tasks, wound healing, neuropathic pain and itch, psychological wellbeing, and return to school or work.

**Conclusions:**

This core outcome set caters for global burn research, and future trials are recommended to include measures of these outcomes.

**What is already known on this topic**

- Outcomes reported in trials of burn care interventions are heterogeneous and limit evidence synthesis

**What this study adds**

- This study shows agreement to seven core outcomes to be reported in all burn care trials

**How this study might affect research, practice, or policy**

- Agreeing outcome measures and implementing the burn core outcome set will enable improved synthesis of research to reduce research waste and to support clinical decision making in global burn care

## Introduction

The provision of evidence to support clinical decision making relies on data from randomised and non-randomised trials to inform systematic reviews.^1 2^ Systematic reviews aim to collate all available high quality empirical evidence to produce conclusions.^3^ One issue that challenges evidence synthesis, is the variation in outcome reporting across trials—outcome reporting heterogeneity.^4 5^ Outcome reporting heterogeneity can be defined as "the reporting of multiple unique outcomes across trials within one healthcare condition,"^6^ and makes an evidence base difficult to synthesise accurately.^7 8^ Burn care systematic reviewers have reported difficulty combining evidence owing to outcome reporting heterogeneity.^9–11^ The limitation is important in burn care—despite high numbers of patients globally (annual incidence 11 million),^12^ clinical uncertainty regarding optimal management persists.

An agreed minimum set of the most important outcomes is needed to standardise, but not restrict, outcome reporting in burn care research through the development of a core outcome set.^5^ A core outcome set is a group of outcomes to be reported in all trials of a specific condition.^5^ These core outcomes are identified scientifically by stakeholders as being the most important in determining the effects of an intervention or treatment.^13 14^ Outcomes need to be relevant to all stakeholders, highlighting a need for joint decision making in outcome choice. Shared decision making is "a process in which clinicians and patients work together to select tests, treatments, management or support packages, based on clinical evidence and the patient’s informed preferences" (https://www.nice.org.uk/Media/Default/About/what-we-do/SDM-consensus-statement.pdf). The impact of shared decision making on outcome quality has been studied.^15 16^ Without similar shared decision making in outcome choice, there is a risk that trial data will be based on outcomes that might not be the most important to patients.

Once a core outcome set is agreed, all future trials of that condition should include the outcome set as a minimum.^13^ Development and use of core outcome sets in burn care trials will simplify evidence synthesis, allowing increased use of research data, increased research relevance, reduced research waste, and ultimately increased evidence based decision making and reduction in clinical practice variation. The aim of this study is to develop an international core outcome set for burn care research using shared decision making.

## Methods

The study ran from April 2017 to November 2019. The Core Outcome Set in Burn Care Research international (COSB-i) was registered on the Core Outcome Measures in Effectiveness Trials (COMET) database (http://www.comet-initiative.org/Studies/Details/798). It has been developed using standards of core outcome sets for development (COS-STAD) and reported in accordance with the Core Outcome Set-STAndards for Reporting (COS-STAR).^17 18^ The protocol has been published.^19^ Three changes to the protocol are explained. The systematic review of clinical outcomes reported in randomised controlled trials of burn care interventions, was registered on PROSPERO (CRD42017060908 https://www.crd.york.ac.uk/prospero/display_record.php?ID=CRD42017060908) and is published.^20^

This core outcome set is intended for use in all research studies comparing interventions for the treatment of adults and children with burns, including surgical and non-surgical management, regardless of the cause or severity of the burn, healthcare setting, or mode of intervention use, and can be used across countries of all World Bank income groups.

Development of COSB-i involved three phases: generation of a comprehensive long list of outcomes informing a survey questionnaire, a Delphi survey involving two questionnaire rounds to gain consensus on the most important outcomes, and a consensus meeting to agree the final core outcome set. The process was overseen by an independently chaired steering group based in the UK. This group comprised of three adult patients with burns and a parent of a child with a burn, two burn researchers, three researchers of core outcome sets, one Cochrane wounds group representative, three burn surgeons, a burn nurse, a psychologist, a occupational therapist, and the UK burn database chair. The committee was chaired by an independent methodologist of core outcome sets (JK).

After discussions with the National Institute for Health Research at a meeting in Bristol in January 2017, it was decided to internationalise the core outcome set, for reasons including the global incidence of burn injury. A broad range of Delphi survey professional participants, from as many disciplines and countries of varying income status as possible, were included.

### Phase 1: generation of a long list of outcomes and a survey questionnaire

Outcomes were identified from three sources^20–22^; a systematic review of clinical outcomes reported in randomised controlled trials, semi-structured interviews with patients and clinical staff, and two published systematic reviews on patient reported outcomes in burn care (table 1).

**Table 1:** Identification of burn clinical outcomes allocated to domains from three information sources

Domain of burn outcome	Count of systematic review clinical outcomes (n=993)	Count of interview outcomes (n=149)	Count of patient reported outcomes (n=45)
Ability to carry out daily tasks	1	9	3
Ability to fight infection	37	—	—
Adherence to treatment	—	—	1
Anxiety about medical procedures	4	1	1
Appearance	3	14	3
Blister fluid	3	1	—
Blood transfusion	11	—	—
Body temperature issues	—	1	1
Bone strength	30	—	—
Breathing and lungs	27	2	—
Bullying	—	4	1
Burden of care for patients or carers	7	15	1
Burn smell	—	1	—
Burn wound healing	144	7	—
Burn wound infection	79	2	—
Burn wound pain	29	4	2
Comfort of dressings	1	2	—
Complications of treatment	52	4	—
Costs of treatment for NHS/hospital	14	—	—
Death from any cause	14	1	—
Death from burn injury	1	—	—
Dignity	—	1	—
Donor site healing	9	—	—
Donor site infection	1	—	—
Donor site outcomes	1	1	—
Donor site pain	6	1	—
Donor site problems after healing	23	—	—
Effect of burn on genes	1	—	—
Effect of burn on how the body uses energy	2	—	—
Effect of scar on movement	3	3	—
Effect on heart and blood circulation	1	—	—
Effects of fluid from a drip	17	—	—
Fitness	12	3	—
Generalised anxiety	1	5	2
Growth after injury	10	1	—
Hair loss	—	1	—
Heart and blood circulation	28	—	—
How well muscles work	9	—	6
Infections other than burn wound infection	7	—	—
Itch	24	3	2
Kidney function	17	—	—
Length of hospital stay	7	4	—
Length of stay in intensive care unit	3	1	—
Length of time on breathing machine	1	—	—
Liver function	11	—	—
Maintenance of body weight	26	—	—
Medical tests to indicate how unwell a patient is	82	—	—
Mental ability	2	1	—
Mobility	22	3	3
More than one organ failing	7	—	—
Muscle strength	30	—	—
Pain during procedures	14	1	—
Personal cost for patient	—	1	—
Psychological wellbeing	1	15	7
Quality and quantity of sleep	17	1	—
Relationships	—	8	8
Return to work or school, or previous function	1	2	3
Scar colour	25	1	—
Scar pain	13	3	—
Scar size	1	—	—
Scar texture	46	1	—
Sepsis	7	—	—
Skin graft healing	21	—	—
Stomach and bowel function	13	5	—
Thirst	—	1	—
Treatment for scars	2	10	—
Understanding of planned care	—	3	—
Use of medicines to treat symptoms	12	1	1

Systematic literature review of clinical outcomes in burn randomised controlled trials were identified by electronic searches of four search engines from January 2012 to December 2016. Searches included trials using medical subject headings and free text terms including "burn," "scald," "thermal injury," and "RCT." Outcomes were classified into domains. Detailed methodology is published elsewhere.^20^

Semi-structured interviews were conducted to identify burn outcomes considered important by multidisciplinary burn professionals and patients or carers. Interviews were conducted with 10 burn care clinical staff (one consultant, three nurses, four therapists, and two others), 14 adult patients, and one child and parent combination, at different times after injury. Sample size was determined by data saturation, with purposive sampling to achieve diversity.^19^ The interviews were audio recorded with consent and transcribed verbatim. Interviews were guided by a topic guide that centred around recovery outcomes affected by healthcare treatment and issues affecting daily life after injury. Analysis used NVivo software. Thematic analysis was conducted by one researcher, with a second researcher reviewing codes and agreeing outcome domains. Domains were defined as broad concepts that group similar outcomes together.^23^

Patient reported outcomes were extracted from two systematic reviews^21 22^ reporting generic and burn specific tools. Most generic measures had been validated with adults from the general population, and were unlikely to be sufficiently sensitive to identify health outcome changes in a burn population.^24^ We decided to use outcome domains from tools for patient reported outcomes related to burns. In the review of patient reported outcomes in child and adolescent burn research, 31 tools were generic, and one was specific to burns. The Children’s Burn Outcomes Questionnaire was designed for patients aged 11-18 years.^25^ In the systematic review assessing patient reported outcomes in adult burn care, 71 tools were generic and six were specific to burns, of which four had been validated in English. BSHS-A is an abbreviated version of the Burn Specific Health Scale assessing quality of life after a burn.^26^ BSHS-B is an abbreviated version of the BSHS-A and the BSHS-R (revised).^27 28^ The Young Adult Burn outcome Questionnaire (YABOQ) measures health outcomes in young burned adults.^29^ BSPAS-A measures anxiety related to pain during or after burn treatment and is a short version of the Burn Specific Pain Anxiety Scale.^30^ We extracted the outcome domains from the five tools.

To develop the questionnaire, outcome domains were initially created from the systematic review of clinical outcomes supplemented by domains from the interviews and data of patient reported outcomes.^20^ Two researchers carried out this process independently and met to discuss the results. A patient and burns nurse assisted in agreeing the outcome de-duplication and subsequent outcome terms extracted from three sources, categorising the outcomes into domains and agreeing domain names. This change to the protocol stated that two patients would undertake this role. The authors and steering group agreed that a research nurse not involved in the project would give independent advice and complement that from the patient. Domains were added to questionnaire items for the survey. The Delphi questionnaire survey was piloted through cognitive interviews with six parents and with adults and young people who reviewed the survey, to assess usability, face validity, and acceptability. The survey was modified as a result of feedback.

We uploaded the final questionnaire survey onto a specifically designed Research Electronic Data CAPture (REDCap) database, with weblinks to the consent, personal characteristics questions, and questionnaire outcome items. REDCap (https://www.project-redcap.org/) is a secure, web based software platform designed to provide an interface for validated data capture.

### Phase 2: prioritisation of outcomes through a Delphi questionnaire survey

Delphi survey participants were multidisciplinary international clinical staff working with burn patients, burn researchers, UK burns patients aged 10 years or older, and carers of children with burns of any age. Patients and carers were identified through four NHS burn services, support groups, and social media. Methods to identify international professional participants included communication via ResearchGate (https://www.researchgate.net/), international professional burn organisations, international burn charities, social media, key country collaborators, and personal email through the authors’ own international contacts.

**Figure 1:**
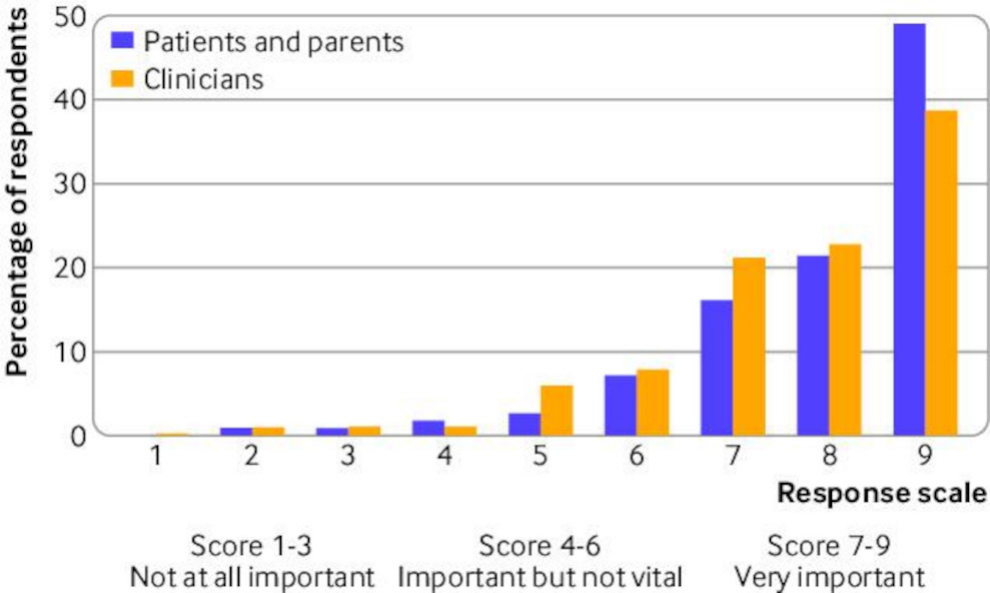
Delphi survey round 2 feedback

Information was provided through a plain English video devised as part of the study (https://www.youtube.com/watch?v=9DYH072uPrQ). For each included questionnaire item (outcome), a 9 point, Likert type response scale was provided (1-3 was not important, 4-6 important but not vital, and 7-9 very important). A zero option was provided to indicate no opinion (eg, no experience of the condition illustrated). Following feedback, we used a coloured traffic light spectrum to facilitate comprehension among young people. At the end of round 1, an option was provided to allow additional outcomes to be suggested. All participants who had completed round 1 were emailed a personalised link to round 2 on the REDCap database. During round 2, participants were shown the distribution of scores for each stakeholder group (alongside the median overall score) for each outcome, with their own score from round 1 and asked to score the outcome again, using the same 1-9 Likert scale and taking this information into consideration (figure 1). No agreed sample size guidelines for numbers of participants currently exist for consensus methods when developing a core outcome set. We aimed to recruit 150 UK patients or carers, and 200 clinical staff and researchers.

Study data were collected and managed using REDCap.^31^ We considered participants to be any individual completing both the consent form and rating at least one survey item. For item ratings, any completed datapoint was included. For each item, the following summary data were produced: number of participants completing; statistical measures (mean and standard deviation, median and interquartile range); and number and percentage of participants rating the item as 1-3, 4-6, and 7-9. Items rated as 0 (no opinion) were excluded from the data. Data were tabulated for the overall sample and according to stakeholder group, whereby participants were grouped as clinical staff (including researchers) or patients/carers.

Predefined criteria for progression of items to round 2 and consensus meeting were agreed by the steering group, were based on previous core outcome work, and were explicit in the protocol.^19^ For progression to round 2, at least 50% of the sample (or of either stakeholder group) needed to rate the item a score of 7-9. More stringent criteria were applied for items to carry through to the consensus meeting; items needed to be rated 8-9 by more than 70% of the participant sample. We assessed attrition between rounds by median and mean survey scores at round 1, comparing those participants who did with did not complete both rounds. Mann Whitney U tests were used. The significance level was set at P<0.05.

### Phase 3: consensus meeting

A consensus meeting was held in London, UK, on 9 October 2019. An independent chair was appointed from a burn research charity (Charlotte Coates, Scar Free Foundation https://scarfree.org.uk/). Clinical staff or patient or carer participants who had completed round 2 of the survey were invited, attending in person or by video conference call. We used online voting software (https://turningtechnologies.com) to enable remote voting. Before voting, a discussion was conducted by the independent chair, to agree questionnaire items (from now on called outcomes) that were similar and could be combined.

Outcomes were voted in or out with real time results shown to participants as a histogram. Two rounds of voting were undertaken. After the first voting round, outcomes with >50% of participants rating it in to include were carried through to the next round. For the second round, a more stringent criterion of >60% of the sample rating it as important to include in the core outcome set (voted in), was used to select items to include in the final set.

### Patient and public involvement

Patients and carers were involved in designing the study protocol and patient information, through participation in the steering group, interviews to inform the long list of core outcomes, participation in the Delphi survey and consensus meeting, and in ongoing dissemination.

## Results

**Figure 2:**
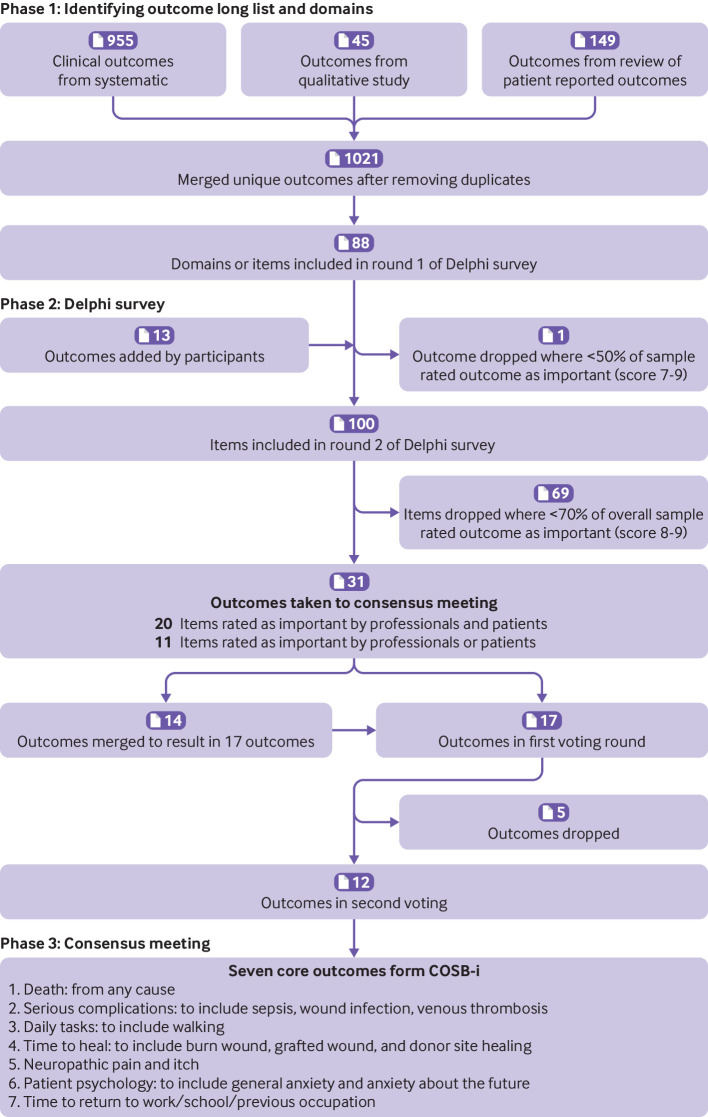
Flow chart for the Core Outcome Set in Burn Care Research international (COSB-i)

A summary of the outcome selection into the final process for the core outcome set is shown in figure 2.

### Phase 1: generation of outcome long list and survey questionnaire

Examination of all data sources identified 1149 outcomes, which after de-duplication left 1021 unique outcomes that were grouped into 68 outcome domains. Additional domains were added for those patient reported outcomes and interview data that did not fit into the existing set of 68. This resulted in 88 questionnaire items (outcomes) constructed for round 1 of the survey.

### Phase 2: prioritisation of outcomes through Delphi questionnaire survey

In round 1 of the survey, 668 (84%) participants were international clinical staff or researchers and 126 (15%) were UK patients or carers. Clinical participants’ personal characteristics for rounds 1 and 2 are in table 2. For round 1, clinical participants originated from 77 countries and five continents. Of these, 120 (18%) came from lower middle and lower income countries. Of the clinical staff, 303 (45%) were doctors, 158 (24%) were allied health professionals, 100 (15%) were nurses, and 88 (13%) were burn researchers. Of the patients, 97 (77%) were adults, 28 (22%) were carers of a child with a burn injury, and one was a young person (11 years) (1%). Eighty (63%) patients were of white British origin, and 52 (41%) had a university education. The mean time since injury in the children of carers who responded was 5.5 years (standard deviation 10.7), and 12.8 years (15.3) for adult patients.

**Table 2:** Personal characteristics of clinical staff participants in Delphi survey rounds 1 and 2

	Survey round 1 (n=668)	Survey round 2 (n=378)
**Clinical staff occupation**		
Doctors		
Consultant in burn care (burn surgeon, plastic surgeon, paediatrician, trauma, pain specialist)	173 (25.9)	87 (23.0)
Anaesthetist or intensivist	84 (12.6)	46 (12.0)
Pathologist	1 (0.2)	0
General practitioner	2 (0.3)	2 (0.5)
Junior doctor or registrar	43 (6.4)	21 (5.6)
Medical student	1 (0.2)	1 (0.3)
Allied health professional		
Burn allied health professional (physiotherapist, occupational therapist, dietician, psychologist, play, speech and language, laser technician, social worker.	158 (23.7)	97 (25.7)
Paramedic	2 (0.3)	2 (0.5)
Nursing		
Burn care nurse, research nurse, or theatre nurse	100 (15.0)	56 (14.8)
Researchers		
Burn researcher	88 (13.2)	58 (15.3)
Other		
Burn charity	1 (0.2)	1 (0.3)
Commercial	1 (0.2)	1 (0.3)
Burn commissioner or service manager	2 (0.3)	2 (0.5)
Medical education	4 (0.6)	2 (0.5)
UK National Institute for Health and Care Research	1 (0.2)	1 (0.3)
Not stated	5 (0.7)	1 (0.3)
**Time spent in burn care**		
6-12 months	45 (6.7)	18 (4.8)
>1-3 years	76 (11.4)	36 (9.5)
>3-5 years	77 (11.5)	44 (11.6)
>5 years	466 (69.8)	278 (73.5)
Not stated	4 (0.6)	2 (0.5)
**World Bank income group**		
High income country	473 (70.8)	306 (81.0)
Upper middle income country	71 (10.6)	32 (8.5)
Low middle income country	95 (14.2)	26 (6.9)
Low income country	25 (3.7)	12 (3.2)
Missing data	4 (0.6)	2 (0.5)

Of all 88 items in round 1, 85 were rated as very important (survey score 7-9) by at least 50% of the sample. Two items did not reach the threshold (thirst, smell of the burn). More than 50% of the patients and carers independently rated these items as very important (7-9), so they were carried through to round 2. One item did not reach the 50% threshold for the overall sample, for either group (mild complications), and was removed. Thirteen new outcomes were suggested and added. One hundred items formed the round 2 survey (figure 2).

Of those participating in round 1, 431 participants (54.3%) undertook round 2. Of all participants in round 2, 53 were patients or carers (42.1% of those completing round 1), and 378 were clinical staff (56.6% of those completing round 1). Details of those completing both rounds, or only round 1, were similar (table 2). Analyses examining differences in outcome ratings between participants who completed round 1 only or rounds 1 and 2 indicated that 24 outcomes were significantly different at P<0.05. However, closer inspection indicated minimal differences between the medians and means for these outcomes; these small differences are unlikely to have substantially affected the outcomes that met the criteria.

Of all the questionnaire items, 31 reached the threshold to be carried through to the consensus meeting, of which 20 had been voted survey scores 8-9 by >70% of all participants combined (ie, clinical staff plus patients or carers). An additional 11 outcomes were voted as more than 8-9 by >70% of either patients or clinical staff. In a change to the protocol, because strong consensus had been achieved through the two Delphi survey rounds, we considered a third survey round to be unnecessary. The raw data and codebook are available through the Dryad repository.^32^

### Phase 3: consensus meeting to agree final core outcome set

The consensus meeting was attended by 28 UK clinical staff and patients, and 19 international clinical staff joined by teleconference. A discussion, chaired independently, was undertaken with participants to determine outcomes that could be pragmatically combined owing to similarity in meaning. Discussion identified overlap between nine outcomes. Agreement was reached to combine specific items; death due to the burn with death from other causes, multiorgan failure and multiorgan dysfunction into organ dysfunction, kidney and lung dysfunction into organ dysfunction, length of stay in intensive care with length of time on a ventilator, anxiety with psychological impact into psychological wellbeing, procedural and background pain into acute pain, and scar pain with itch into neuropathic pain, to group specified complications under one heading with the inclusion of sepsis, wound infection, and thrombosis, and to incorporate burn wound, grafted wound, and donor site healing into burn wound healing. Combining outcomes resulted in 17 outcomes on which to vote.

Of the 17 outcomes voted in on round 1, outcomes with at least 50% of participants stating that they should be included were identified, resulting in five outcomes being removed before round 2. Of the 12 items voted on in round 2, seven reached the criteria to be included in the final core outcome set. These seven outcomes were agreed to represent the final set for burn care research and are shown in box 1.

Box 1 **Final Core Outcome Set in Burn Care Research international (COSB-i)**

- Death from the burn or any cause
- Prespecified serious complications or adverse events; for example, sepsis, wound infection, and thrombotic events
- Ability to do daily tasks
- Time to wound healing, including that of grafted and donor site wounds
- Long term (after healing) neuropathic pain and itch
- Psychological wellbeing
- Time to return to work, school, or previous occupation

## Discussion

### Principal findings

International burn care is inconsistent, resulting in varying healthcare outcomes. Increased, high quality, synthesised evidence to support decision making is needed, but hindered by variation in outcome reporting. This study has developed a core outcome set to standardise, but not restrict, outcome reporting in burn care trials. It was developed using shared decision making by UK patients/carers and international clinicians/researchers, using novel strategies to achieve consensus, including a web based survey to ensure engagement. The core outcome set was generated from three information sources informing a modified Delphi survey. Consensus meeting voting generated seven core outcomes.

Funding bodies are advocating the use of core outcome sets, and their uptake among triallists is increasing.^33^ New core outcome sets are increasingly being developed in specialties such as dermatology, rheumatology, paediatrics, and breast and colorectal surgery.^8 34–39^ These core outcome sets are more commonly developed using international participants, to increase dissemination, to support applicability in global healthcare settings, and to make it more likely that the core outcome set will be used wherever future trials take place.^40–42^ The core outcome set in this study included 794 survey participants, of whom 668 were international clinical staff or researchers, from 77 countries of all four world income groups; 18% were from low income countries. This diversity is important for the global relevance of a core outcome set for burn injury.^43–46^

Burn injury is a form of trauma. The outcomes chosen are similar in type to those agreed in other trauma related core outcome sets. In a core outcome set for traumatic dental injuries, outcomes include healing, pain, complications, functional status of teeth, and quality of life including return to work.^47^ The COSB-i outcomes are also similar to the core outcomes chosen for trials of hip fracture interventions; mortality, pain, activities of daily living, mobility, and quality of life. Participants for the core outcome set for whiplash injury agreed on six core outcomes: physical function, perceived recovery, work and social functioning, psychological functioning, quality of life, and pain.

### Strengths and limitations of the study

The outcomes chosen for this core outcome set will reflect priorities for all stakeholder groups. Implementation will result in more effective dissemination, a more meaningful result for international research, and an emerging shared decision making network researching outcomes for burn care.^48^ Shared decision making has a traditional definition. The term could also imply joint decisions in research outcome choice, weighing the views of all stakeholders equally.

Outcomes achieved with clinicians alone often focus on short term physiological endpoints that are often of less importance to patients. In this study, stakeholders agreed on outcomes spanning short and long term recovery. Acute complications including infection and healing time are outcomes to measure the effect of interventions in burn efficacy trials. The other outcomes, (death, ability to undertake tasks of daily living, neuropathic pain and itch, psychological wellbeing, and time to return to work or school), are more likely to be of value when assessing clinical interventions in longer term pragmatic trials. The participant inclusivity and co-production is increasingly common in the development of core outcome sets, and is a strength of this study.^40 49^

Further study strengths lie in the comprehensive search for potential outcomes, through three sources, including patient reported outcomes. Some outcomes in the final core outcome set (eg, daily activities; return to school, work, or previous occupation) were not commonly reported in previous systematic reviews of patient reported outcomes, adding to the likelihood that new knowledge will be obtained by using this current set. Our study followed the standards for development and reporting of core outcome sets and previous practice endorsed by the COMET initiative. Three methodology changes from the published protocol have been explained.

A limitation of the study was the lack of representation from international patients, which could have limited the generalisability of the study to international patients. Recruiting global patients incurs costs and time for questionnaire translation and validation, along with ethical research permissions to achieve in many countries. Such desired recruitment was impossible owing to time and financial constraints in this study. The study also had a lack of representation of children, although carers of children were included.

Future work will require operationalising the core outcome set with clearly defined outcomes, outcome measures, agreed measurement timepoints and a method to update the list to reflect changes in knowledge. Ultimately, reporting data for these core outcomes will make burn trial design more relevant and make the ability to synthesise evidence more effective with reduced research waste. We recommend that the COSB-i core outcome set is used consistently in burn care research.

### Conclusions

This study used rigorous methodology and international shared decision making to agree a minimum set of core outcomes to be reported in trials assessing burn care interventions. The development of this core outcome set was undertaken to promote the standardised reporting of outcomes and facilitate the robust evaluation of burn care. We recommend that future trials include measures of these seven outcomes.

## Data Availability

Data are available in a public, open access repository. Additional data available through Dryad repository: doi:10.5061/dryad.79cnp5htr.
